# Preparation, Characterization, and Lubrication Performances of Water-Based Nanolubricant for Micro Rolling Strips

**DOI:** 10.3390/ma17020516

**Published:** 2024-01-21

**Authors:** Yuchuan Zhu, Hongmei Zhang, Na Li, Zhengyi Jiang

**Affiliations:** 1 School of Materials and Metallurgy, University of Science and Technology Liaoning, Anshan 114051, China; lilyzhm68@163.com (H.Z.); lina@ustl.edu.cn (N.L.); 2 School of Mechanical, Materials, Mechatronic and Biomedical Engineering, University of Wollongong, Wollongong, NSW 2522, Australia

**Keywords:** Fe_3_O_4_ nanoparticles, water-based lubricant, tribological properties, rolling test, processing quality

## Abstract

Water-based nanolubricants are widely used in rolling processes due to their unique characteristics. As a common additive, nanoparticles could significantly improve the tribological properties of the lubricant. However, the effect of the physical properties of the particles on the anti-friction behavior is unclear. In this study, the effect of Fe_3_O_4_ nanoparticles as an additive for the prepared lubricant is studied. The tribological properties of Fe_3_O_4_ water-based nanolubricant are examined using a tribometer and a scratch meter. The absorption energy is calculated using the molecular dynamic simulation method, and the best parameters for the preparation of the nanolubricant are obtained. The developed nanolubricant is used in the rolling process. The results show that the processing quality of samples is promoted and the tribological properties of water-based lubricant can be significantly promoted by an Fe_3_O_4_ nanoparticle additive. An economical and environmentally friendly method is presented through which the water-based Fe_3_O_4_ nanolubricant can be prepared for the replacement of oil-based lubricant in cold rolling strips.

## 1. Introduction

In recent years, water-based lubricants have been used in metal working processes. The environmental and cost-saving characteristics are favored by the majority of suppliers and users. The development of metal-forming lubricants is a very long process. The techniques required for the making of water-based lubricants have been and continue to be studied by many researchers [1]. With the development of nanotechnology, different varieties of nanoparticles are added to improve the performance of lubricants [2]. Nanoparticles of metals [3], metallic oxides [4,5], non-metallic oxides [6,7,8,9], sulfides [10,11], composites [12,13,14], diamonds [15,16], graphene oxides [17,18,19], and rare earth materials [20] are dispersed in the lubricant. Both size and concentration of nanoparticles are important parameters for the preparation of nanolubricants [7,9,21]. The anti-friction and anti-wear performance of the lubricant is the most important parameter. It is reported that the tribological performance of lubricants could be improved by mixing some smaller nanoparticles [21]. Although the anti-friction and anti-wear performance of lubricants has been shown to decrease as a result of high concentrations of nanoparticles [9], nanoparticles also increase the viscosity of the lubricant. The viscosity has a significant effect on the performance of lubricants [22,23,24].

It is important that nanoparticles are stably dispersed in water-based lubricants, with amphiphilic surfactants usually used to improve the dispersion of nanoparticles in solution [2]. The dispersion of TiO_2_ nanoparticles in water-based lubricants is improved by polyethyleneimine (PET) [25,26]. PET is a water-soluble surfactant which can be well dissolved in the nanoparticles, and stably dispersed in water-based solutions as the mixing medium. In 2017, sodium dodecyl sulfate was used in vegetable lubricants to improve the dispersion of CeO_2_ and plotetrafluoroethylene (PTFE) nanoparticles [20,27]. In 2018, it was found that the addition of stronger ammonia water to the modified silica nanoparticles could improve the dispersion stability of the nanoparticles in the solution [28]. In 2019, a solution containing certain concentrations of ethanol, tetraethyl orthosilicate, and ammonia was prepared to modify the surface of silica nanoparticles [29]. In 2020, a further study used sodium dodecyl benzene sulfonate (SDBS) to modify the surface of MoS_2_ nanoparticles. As a result, the tribological performance of water-based solutions significantly improved [11]. The TiO_2_ nanoparticles modified by the SDBS were stably dispersed in a water-based lubricant, with polyacrylic acid sodium salt (PAAS) used as a thickening agent to improve the tribological performance of the prepared lubricant [30]. A wide variety of surfactants have been used thus far to modify different nanoparticles. However, the molecular dynamics of the surfactant on the surface of nanoparticles and the friction pair remain unclear.

A large amount of iron oxide scales is produced in the hot rolling line every year, with the stacking and pouring of significant quantities of iron oxide scales causing environmental problems. The recycling of iron oxide scales would solve this problem. The basic ingredient of iron oxide scales is Fe_3_O_4_. New research suggests that oxide nanoparticles can improve the performance of rolling lubricants. A new method for the recycling of iron oxide scales has been proposed. The Fe_3_O_4_ nanoparticles are prepared from the iron oxide scales, and used as a lubricant additive, improving the performance and quality of the rolling lubricant.

Previous studies have shown that the tribological performance of industrial lard could be significantly improved by adding Fe_3_O_4_ nanoparticles [31]; however, oil-based nanoparticle lubricants prepared with industrial lard as the based suspension would cause some problems. Under the high working temperature, oil-based lubricants can easily produce some poisonous gases and pollute the environment. The raw material required for the production of oil-based lubricants is also highly costly. In this study, a water-based nanolubricant was prepared to replace the oil-based lubricant in cold rolling strips; the environment and cost problem would, therefore, be solved.

Nanosized particles of Fe_3_O_4_ were added to the water-based lubricants. The lubricants, which contained nanoparticles of different diameters, were tested in the four-ball tribometer. The anti-friction and anti-wear performances of the prepared lubricants were examined using a scratch meter under different nanoparticle concentrations. To test the molecular dynamics theory, the surfactants effect on the absorption energy of a water-based solution was analyzed. At last, the prepared water-based nanolubricant was applied to the four-high micro rolling mill. The copper strips were processed in the four-high rolling mill. The processing quality of the samples was analyzed using a surface roughness meter and a microscope. The performance of the prepared water-based nanolubricant was studied.

## 2. Experimental Design

The water-based Fe_3_O_4_ nanoparticle lubricant was prepared by ultrasonication and water bath. The friction properties of the water-based nanolubricant were measured using a four-ball tribometer and a scratch tester. The absorption energy of the water-based nanolubricant was calculated through the implementation of molecular dynamics. Subsequently, the water-based Fe_3_O_4_ nanoparticle lubricant was added to the lubrication system of the four-high rolling mill. The roll forces were obtained under different lubrication conditions and the surface profile of rolled samples was measured using a surface roughness meter. It is proved in this study that the prepared lubricants have excellent tribological properties and lubrication.

### 2.1. Preparation of Lubricants

**Figure 1:**
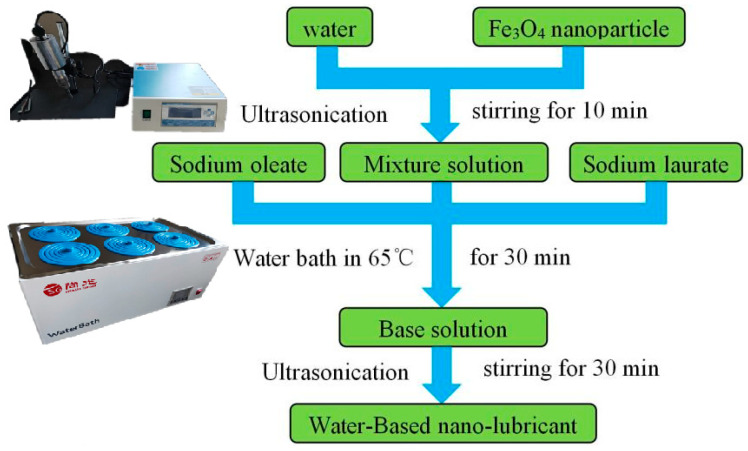
The preparation of water-based nanolubricants.

A new method for the preparation of water-based Fe_3_O_4_ nanolubricants is shown in Figure 1. The Fe_3_O_4_ nanoparticles were mixed with deionized water. The ball shape of nanoparticles was selected; other shapes of nanoparticles were not considered as part of this study. In general, nanoparticles tend to easily agglomerate. In this study, many larger scale particles were dispersed in solution. After stirring for 10 min, the agglomerated particles were destroyed using ultrasonication. Some surfactants were added to the solution to prevent particle agglomeration. Sodium oleate and sodium laurate were added following a ratio of oleate acid to laurate acid of 2:1. After the surfactants were fully dissolved, the solution was immersed in a bath of constant temperature for 30 min. The surfaces of nanoparticles were adequately coated with surfactants. The base solution was stirred for 30 min by ultrasonication. The zeta potential of different water-based lubricants was measured. The zeta potential of a 10 wt% water-based nanolubricant is −40.45 mV. As a result, the nanoparticles are stably dispersed in solution, and the water-based nanolubricant is prepared for the rolling test.

### 2.2. Tribological Tests

**Figure 2:**
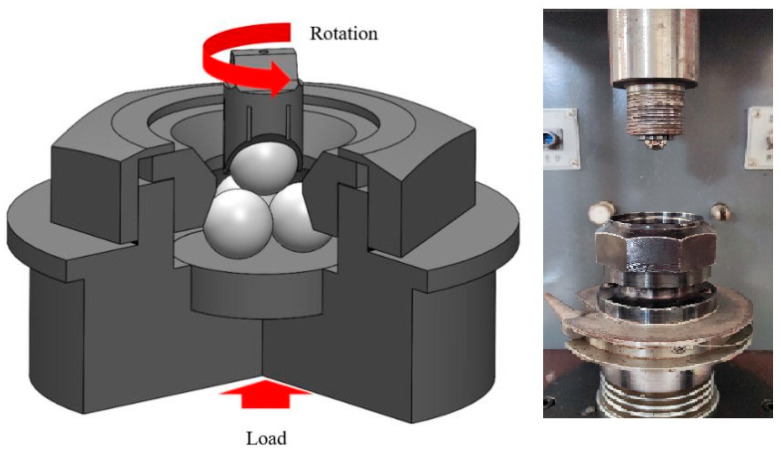
Four-ball tribometer [31].

The four-ball tribometer and scratch tester were used to measure the performance of the nanolubricant. An illustration of the structure of the four-ball tribometer is shown in Figure 2. The test was conducted in the micromolding laboratory at the University of Science and Technology Liaoning. There were four steel balls in the testing area. One steel ball was turned by a spindle at a constant speed, while the others touched the rotating ball at a constant load. The tested lubricants were poured into the testing area and the four steel balls were immersed in the tested lubricants. The test load of the four-ball tribometer was 60 N to 10 kN. The friction force was 0 N to 300 N. The spindle speed was 200 r/min to 2000 r/min. The working temperature ranged from room temperature to 75 °C. The four-ball tribometer was produced by Jinan Star Test Technology Co., Ltd. (Jinan, China). The maximum non-seizure load and friction performance are important indicators of rolling lubricants. The spindle speed was set to 1450 r/min and the load was set to range from 98 N to 1961 N. After a 10 s test, the wear scar diameter of the steel ball was measured under a microscope. The maximum non-seizure load of lubricants (P_B_) was measured. The coefficient of friction (COF) and friction force (F) were obtained after a long-time wear test. In the test, the rotation velocity of the upper ball was set to 1200 r/min, the constant load was 392 N, and the testing time was 30 min. The COF and F were measured to verify the friction reduction and anti-wear behavior of the lubricants. The wear scar diameters of the bottom balls were measured using a microscope.

**Figure 3:**
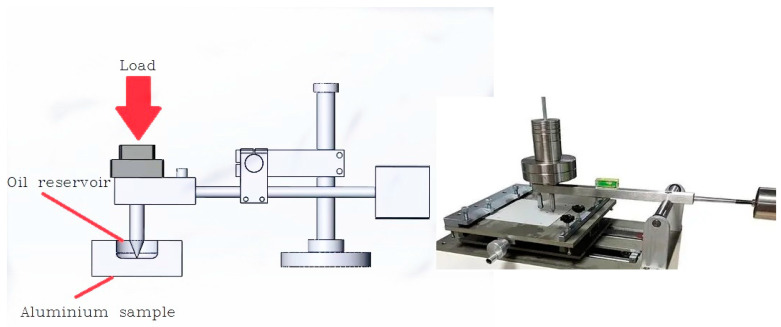
Scratch tester.

An illustration of the structure of the scratch tester is shown in Figure 3. The aluminum pallets were prepared to examine the friction performance of the water-based nano-lubricant. The pallet was fixed to the test area. The probe made the contact with the bottom of the pallet without any load. The testing equipment of the scratch tester was set to zero. The tested lubricant was poured in the bottom of the pallet. A weight of 100 N was exerted on the probe. The constant load was 100 N. The pallet was moved a short distance at a low speed in the direction perpendicular to the load. The friction force was measured using the testing equipment for 20 min. The morphology of the tested pallet was observed by microscope. The friction performance of water-based lubricants was revealed by the friction force and the morphology of the pallet.

### 2.3. Rolling Test

The four-high reversing micro rolling mill was used to examine the performance of the water-based nanolubricant. The diameter of the backup roll was 120 mm. The diameter of the work roll was 30 mm. The effective roll width was 80 mm. The maximum allowable rolling force was 500 KN. The maximum velocity achieved by the rolling mechanism was 2 m/min. The copper sample was processed by a four-high rolling mill under different lubricating conditions. The thickness of the copper strips was 0.2 mm. The width of the copper sample was 30 mm. The surface roughness of the rolled sample was measured under different rolling forces using a surface roughness meter. The morphology of the rolled sample was observed using microscope. The lubrication performance of the nanolubricants was determined from the tested data.

### 2.4. Numerical Simulation

**Figure 4:**
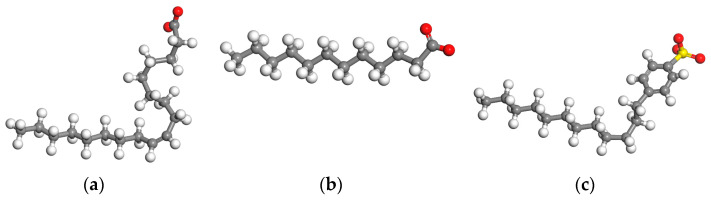
The molecular structure of tested ingredients. (**a**) oleinic acid [31]. (**b**) lauric acid. (**c**) SDBS.

The adsorption energy of lubricants is an important property. The higher the adsorption energy, the more stable the adsorption by the lubricant on the surface of friction pairs. The adsorption energy of lubricants containing different additives was calculated by numerical simulation. The latter is based on the theory of molecular dynamics and the adsorption energy was determined under different working temperatures using Materials Studio. The force field was set to COMPASSⅡ. The molecular structures of the tested ingredients were used in molecular dynamics calculation. The oleinic acid is shown in Figure 4a, the lauric acid is shown in Figure 4b, and the SDBS is shown in Figure 4c. The lattice constant of the copper surface was set to [0,0,1]. The lattice constant of Fe_3_O_4_ was set to [0,0,1]. The color of different atoms is showed in Table 1.

## 3. Results

### 3.1. Tribological Properties

#### 3.1.1. Results of the Four-Ball Tribometer

**Figure 5:**
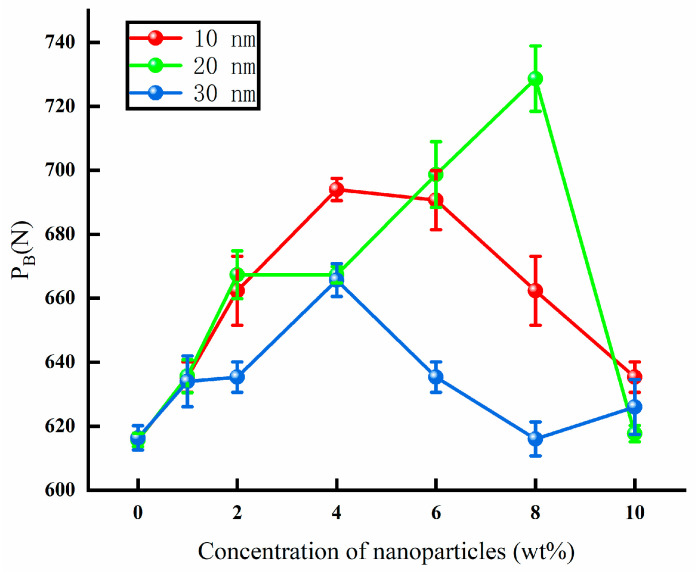
The maximum non-seizure load of lubricants (P_B_) under different concentrations of mixed particles.

The maximum non-seizure load of the water-based nanolubricant (P_B_) is shown in Figure 5. The base solution only containing some additives was tested several times. The value of P_B_ was equal to 618 N. Following addition of the nanoparticles to the base solution, the P_B_ almost certainly increased. The concentration of particles has a great effect on the P_B_ of a lubricant. When a low concentration of nanoparticles is added to a lubricant, the continuity of solution is reduced. By increasing the concentration of the added nanoparticles, the continuity is repaired and the film-forming capacity of the lubricant improves. However, with increasing nanoparticle concentrations there is a higher risk of agglomeration, thus leading to a decrease in the film-forming capacity of the lubricant. When the diameter of the particles was 10 nm, the P_B_ improved, with the concentration increasing to 4 wt%. The maximum P_B_ of the lubricant containing nanoparticles with a diameter of 10 nm was obtained, with the concentration of particles reaching 4 wt%. When the concentration of the mixed particles was continuously increased, the P_B_ suffered a reduction in the increase of the concentration. The P_B_ of the lubricant containing a 10 wt% concentration of 10 nm particles was more than the P_B_ of the base solution. When the diameter of the nanoparticles was 20 nm, the P_B_ of the lubricant significantly improved. The maximum P_B_ of the lubricant was associated with a concentration of 8 wt%. When the concentration increased to 10 wt%, the P_B_ of the lubricant decreased significantly. The P_B_ of the nanolubricant was equal to the P_B_ of base solution. When the diameter of the nanoparticles was 30 nm, the P_B_ certainly improved. The effect of concentration is almost the same with the different concentrations of 10 nm particles. The maximum P_B_ of the lubricant was obtained when the concentration was stabilized at 4 wt%. The P_B_ significantly decreased when the concentration increased to 8 wt%. The P_B_ of the lubricant containing a 10 wt% particle concentration certainly improved. Therefore, the addition of nanoparticles appears to have some positive effects on the maximum non-seizure load of the lubricant. The film strength of the lubricant can be improved by a certain concentration of nanoparticles.

**Figure 6:**
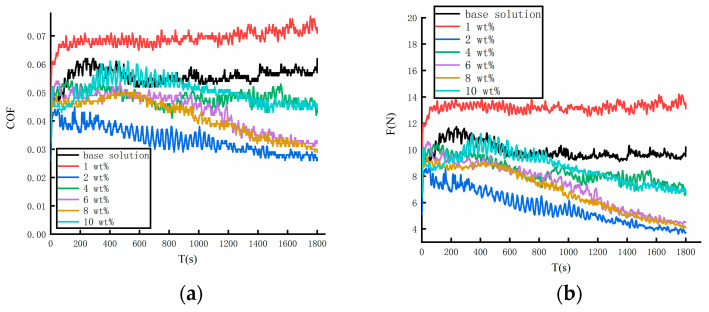
The coefficients of friction and friction force during the long-time test. (**a**) Coefficients of friction. (**b**) Friction force.

The COFs of different lubricants were measured using a four-ball tribometer for 30 min. The COF and the friction force are shown in Figure 6a,b. The friction behavior of different lubricants was discovered by the COF. When the diameter of mixed particles was 20 nm, the film strength of water-based nanolubricants exhibited a significant improvement. Therefore, lubricants containing different concentrations of 20 nm particles were used for the long-time friction–wear test. The COFs of the base solution ranged from 0.045 to 0.06 during the test. When lower concentrations of particles were mixed in the base solution, the COF significantly increased. The particles break the flow properties of lubricants, causing the anti-friction performance of lubricants to suffer a reduction. The COF decreases with increasing particle concentrations and, as a result, the anti-friction performance of the lubricants experiences an improvement. When the concentration of nanoparticles was 2 wt%, the value of COF reached its minimum. With increasing particle concentrations, the anti-friction performance of the lubricant suffers a breakdown. However, the COF of the lubricants containing 6 wt% particle concentrations was the same as that of the lubricant containing an 8 wt% particle concentration. The anti-friction performance of lubricants containing 6 wt% and 8 wt% were found to be excellent after the long-time test.

**Figure 7:**
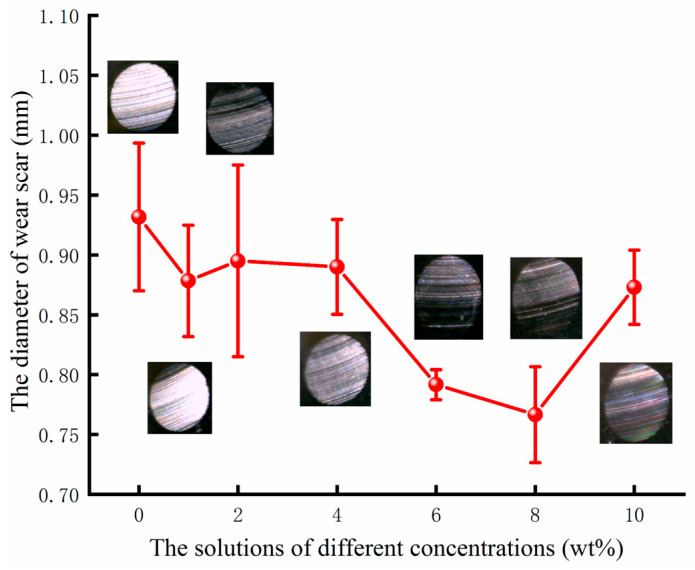
The diameter of the wear scar under different lubricants.

The morphology of the wear scar also indicates the anti-friction performance of the water-based lubricant. The value and wear scar are shown in Figure 7. The diameter of the wear scar suffered a reduction with increasing concentrations of particles. When the particle content of the lubricant was 6 wt%, the diameter decreased significantly. When the concentration increased to 8 wt%, the diameter reached its minimum in several experiments. However, when the concentration increased to 10 wt%, the average diameter significantly increased. The performance of the lubricant can also be analyzed based on the wear scar. After the long-time tests, the wear scar was more obvious and the anti-friction performance of the lubricant became worse. The wear scar in the testing base solution was the most obvious. Following the mixing of the nanoparticles, the wear scar tended to change. It appears fuzzier when the concentration of particles reached 6 wt% or 8 wt%. In conclusion, when the water-based lubricants are mixed with particles in 6 wt% or 8 wt% concentrations, the anti-friction performance of the lubricant seems to improve. To validate these test results, the lubricants were further tested in a scratch tester.

#### 3.1.2. Results of Scratch Tester

**Figure 8:**
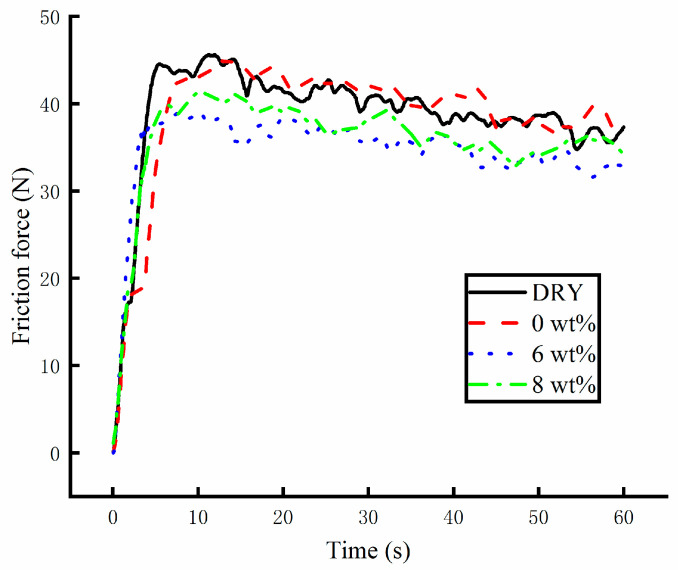
The friction force recorded during the scratch test under different lubricating conditions.

**Figure 9:**
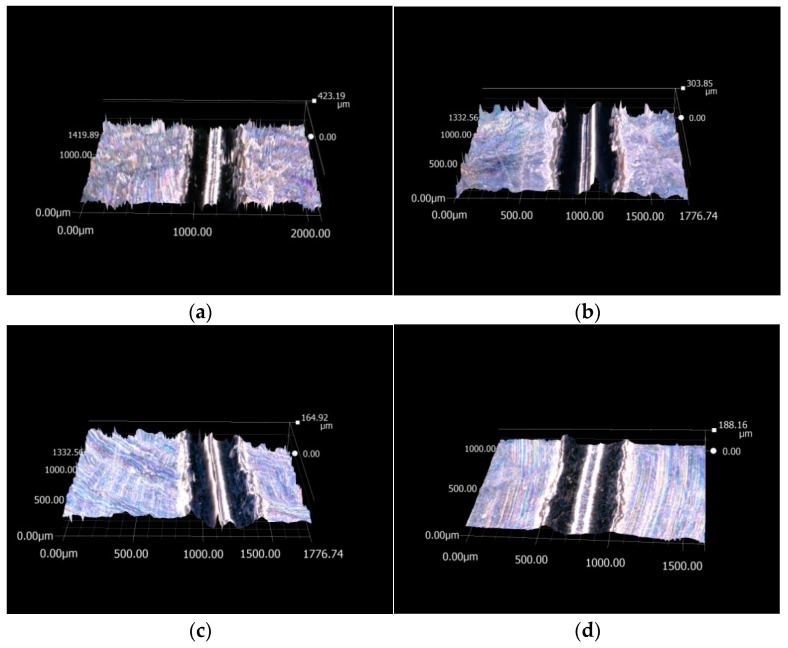
The morphology of the scratch under different lubricating conditions. (**a**) Dry friction condition. (**b**) Base solution. (**c**) Water-based nanolubricant (6 wt%). (**d**) Water-based nanolubricant (8 wt%).

The base solution and the water-based lubricants were tested in a scratch tester. The friction force between the probe and the pallet was recorded under different lubricating conditions. The load was set to 100 N. The friction force of the scratch test under different lubricating conditions is shown in Figure 8. The friction force quickly increased at the beginning of the tests. The maximum friction force was obtained after about 5 s. The test system run stably after 5 s. Subsequently, the nanolubricant flow over the surface of the friction pair caused the friction force to decrease over time. Prior to the system running stably, the friction force without any lubricant was similar to that of recorded under other lubricating conditions. The difference in the friction force was found after 5 s. When the water-based Fe_3_O_4_ nanolubricant was applied to the system, the friction force of the friction pair decreased. The nanoparticles with surface defects in the test samples and the film-forming capacity of the lubricant experienced a marked improvement following addition of Fe_3_O_4_ nanoparticles. In the test of the 6 wt% water-based nanolubricant, the lowest friction force was measured. In the test of the 8 wt% water-based nanolubricant, the friction force slightly increased. For the base solution test, the recorded friction force was the same as that caused by complete absence of lubricant solutions. The morphology of the pallet is shown in Figure 9. The surface morphology measurement of the scratch was performed with a microscope. The rough surface of the scratch was observed under dry friction conditions. The surface roughness of the scratch improved following usage of the base solution. The better surface profile of the scratch was achieved using the 6 wt% water-based nanolubricant. The nanoparticles can be seen in the scratch. When the 8 wt% water-based nanolubricant was tested, the surface quality worsened slightly.

### 3.2. Simulation Calculation of Absorption Energy

#### 3.2.1. Absorption Energy of Base Solution

**Figure 10:**
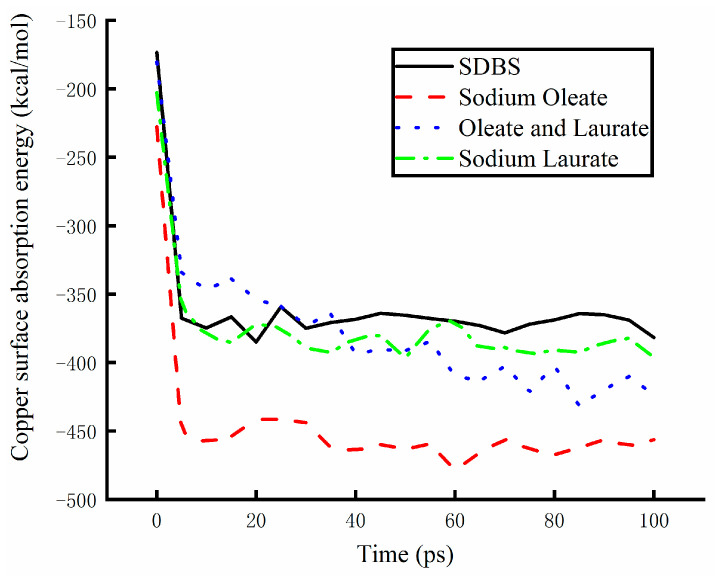
The absorption energy of base solutions following addition of different surfactants.

Absorption energy has a great effect on the performance of lubricants. The absorption energy of solutions obtained following the addition of different additives was calculated using a numerical simulation method. It is based on the molecular dynamics, and the absorption energy of solutions containing different additives was measured at different temperatures. The best additive was used to prepare the water-based lubricants. The absorption energies of the base solutions are shown in Figure 10 at a temperature of 498 K. The copper samples were processed on the cold rolling mill. The working temperature ranged from 298 K to 498 K. The highest temperature was set as the limiting temperature of the simulation. The absorption energy of the tested solutions exhibited great differences at the limiting temperature. The SDBS is a common surfactant. The water-based solution prepared with the addition of SDBS demonstrated a stable absorption capacity. When the base solution was prepared with sodium laurate instead of SDBS, the absorption capacity improved slightly. When the surfactant was replaced by oleate or laurate, the absorption capacity significantly improved after 40 ps. The best absorption capacity recorded for a solution containing the sodium oleate was measured. The absorption capacity of a water-based solution can be effectively improved by the addition of sodium oleate at the limiting temperature.

**Figure 11:**
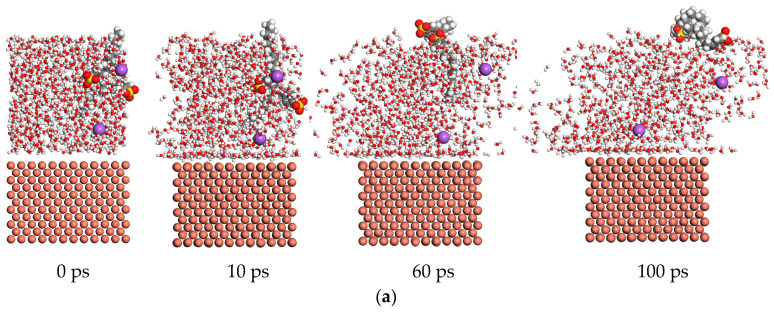

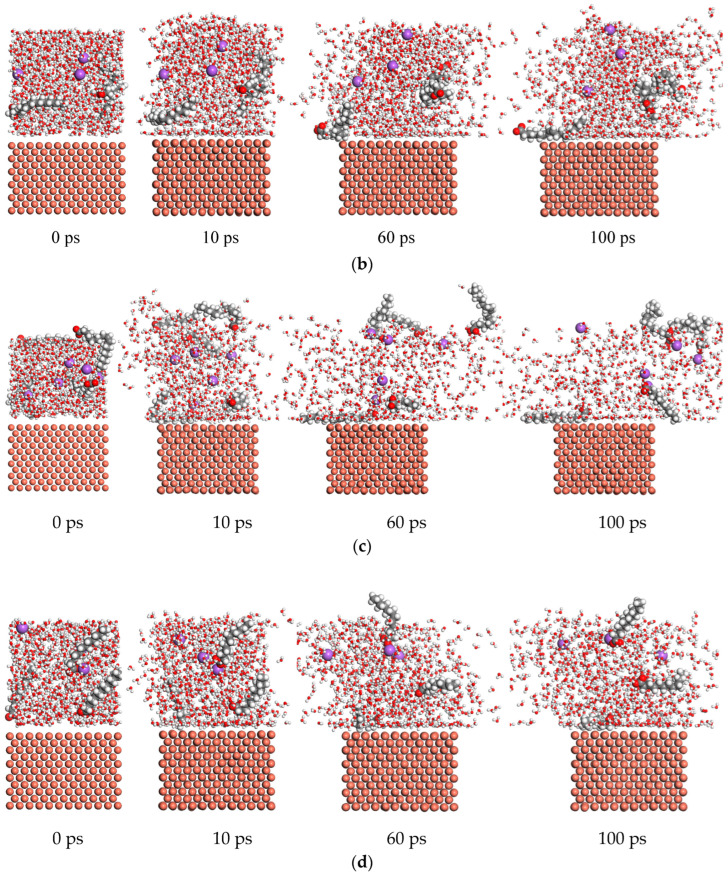
The dynamic path molecules of different base solutions. (**a**) The dynamic path molecules of the base solution mixed with SDBS. (**b**) The dynamic path molecules of the base solution mixed with sodium oleate. (**c**) The dynamic path molecules of the base solution mixed with sodium oleate and sodium laurate. (**d**) The dynamic path molecules of the base solution mixed with sodium laurate.

The dynamic details of different additive molecules are shown in Figure 11 at different times. The dynamic path of molecules was recorded at 498 K. In Figure 11a, the SDBS is prepared for the base solution, and the molecules of SDBS keep a certain distance from the surface of Cu. Throughout the whole time period, the absorption energy is provided by many molecules of water. Therefore, the absorption power of the base solution to which SDBS is added maintains a stable level. In Figure 11b, the long-chain molecules of the sodium oleate are close to the surface of Cu. The absorption energy is almost provided by the molecules of additives. When the sodium oleate is added to prepare the base solution, a better absorption power is obtained. In Figure 11c, the sodium oleate and sodium laurate are mixed into the base solution, and the long-chain molecules have a great effect on the dynamic path of water molecules, with part of the water molecules attracted and driven away by the surface of the copper. Therefore, the absorption power of the mix base solution is weaker than that of the base solution obtained through addition of sodium oleate. The sodium laurate was prepared for the base solution as shown in Figure 11d. At 10 ps, the molecules of the sodium laurate began to come into contact with the surface molecules of the copper sample. At 100 ps, a whole-chain molecule of the sodium laurate was adsorbed by the molecules of copper. However, the absorption energy of the sodium laurate molecules was lower than that exhibited by the sodium oleate molecules, and the absorption energy of the base solution to which sodium laurate was added was weaker than that exhibited by the sodium oleate.

**Figure 12:**
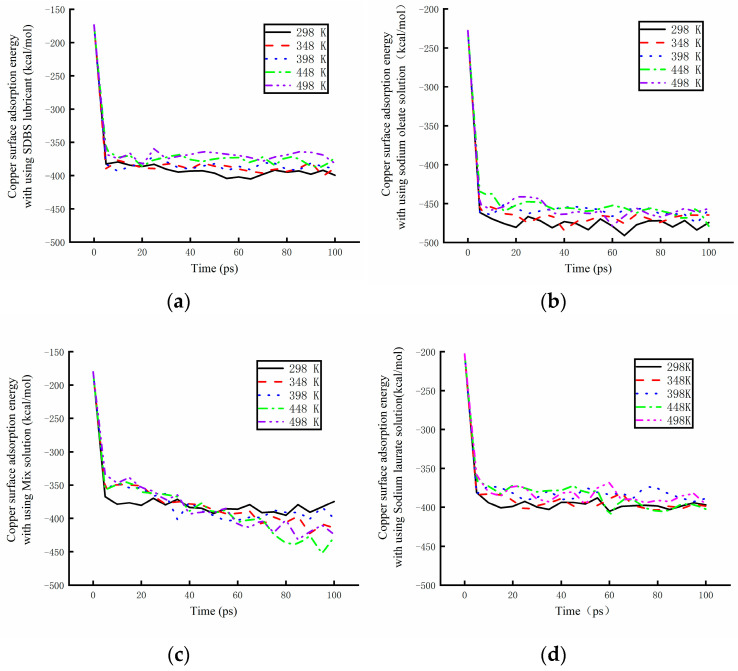
The absorption energy of different base solutions under different working temperatures. (**a**) Absorption energy of the SDBS solution. (**b**)Absorption energy of the sodium oleate solution. (**c**) Absorption energy of the mix solution. (**d**) Absorption energy of the sodium laurate solution.

The absorption energy of different base solutions is shown in Figure 12 under different working temperatures. The effect of the environment temperature was researched using a numerical simulation method. As it can be seen from Figure 12a, the absorption energy of the base solution mixed with SDBS became larger with increasing environmental temperatures. The absorption power was found to be weaker at high temperatures. The working temperature was also found to have some effects on the absorption energy of the base solution mixed with sodium oleate, as shown in Figure 12b. The absorption power reached its maximum at 298 K. With an increase of 50 K, the absorption energy experienced almost no change. However, when the temperature increased to 398 K, the absorption energy became higher, before experiencing a weakening at higher working temperatures. Figure 12c shows that the working temperature has a significant effect on the absorption energy of the base solution mixed with sodium oleate and sodium laurate. When the working temperature increased, the velocity of the molecules in solution also increased. The molecules of surfactants became able to easily reach the copper surface. The absorption energy of the solutions became unstable at high temperatures. With the increase in the working temperature, the value of the absorption energy decreased at different rates. At 448 K, the rate reached its maximum. The best absorption power was obtained after 100 ps. When the temperature rose to 498 K, the absorption energy increased by a lower amount. As shown in Figure 12d, the absorption energy of the solution mixed with sodium laurate was determined by numerical simulation under different temperatures. The energy was found to increase with increasing working temperatures. The absorption power became weaker at the high temperature.

#### 3.2.2. Absorption Energy of the Water-Based Nanolubricant

**Figure 13:**
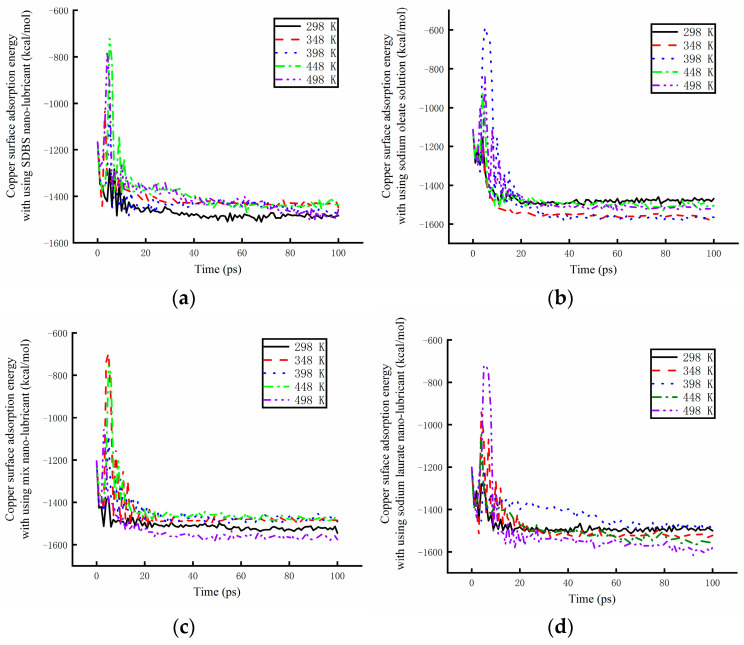
The absorption energy of different lubricants mixed with Fe_3_O_4_ nanoparticles. (**a**) Absorption energy of the SDBS nanolubricant. (**b**) Absorption energy of the sodium oleate nanolubricant. (**c**) Absorption energy of the mix nanolubricant. (**d**) Absorption energy of the sodium oleate nanolubricant.

The absorption energy of the base solutions was studied by mixing different additives. When the Fe_3_O_4_ nanoparticles were stably dispersed within the base solution, the absorption energy of the solution significantly improved. In Figure 13, the absorption energy of water-based Fe_3_O_4_ nanolubricants is shown under different temperatures. The best absorption energy of base solutions was found to equal −500 kcal/mol. When the nanoparticles were mixed in the solution, the absorption energy greatly improved. The value of the absorption energy decreased to −1400 kcal/mol. The absorption energy of the nanoparticles added to the SDBS solutions is shown in Figure 13a under different working temperatures. The absorption energy was unstable until 10 ps. At 298 K, the value of the absorption energy reached its minimum. With increasing working temperatures, the absorption energy also increased: the value of the absorption energy became higher, and the absorption power became lower. Therefore, the absorption power of the lubricant mixed with SDBS decreased on the surface of the copper sample at higher working temperatures. When the base solution was obtained by mixing sodium oleate and water, the absorption energy of the lubricant exhibited differences relative to the SDBS lubricant, as shown in Figure 13b. After 20 ps, the absorption energy became stable. At 298 K, the value of absorption energy reached its maximum. The absorption energy decreased with increasing working temperatures. The absorption power of the sodium oleate solution increased at high working temperatures. The absorption energy exhibited by the solution obtained following the simultaneous addition of sodium oleate and sodium laurate is shown in Figure 13c under different temperatures. The absorption energy was lower than that of the other solution at 298 K. The nanolubricant containing the sodium oleate and sodium laurate achieved its best stable absorption power at 298 K, with the absorption energy only experiencing a slight change with the increase of the working temperature. At 498 K, the energy significantly decreased, with the solution acquiring, therefore, more absorption power. The absorption energy of the sodium laurate solution is shown in Figure 13d. After 20 ps, the absorption energy leveled off. However, at 398 K, the absorption energy reached a special rate. The molecules of surfactants were easily captured by Fe_3_O_4_ nanoparticles at 398 K. More molecules of water reached the surface of the copper sample. The value of the absorption energy increased, while the absorption power became weaker than that under other working temperatures. Therefore, following the addition of nanoparticles to the sodium laurate solution, the absorption power became unstable under different temperatures on the surface of the copper sample.

**Figure 14:**
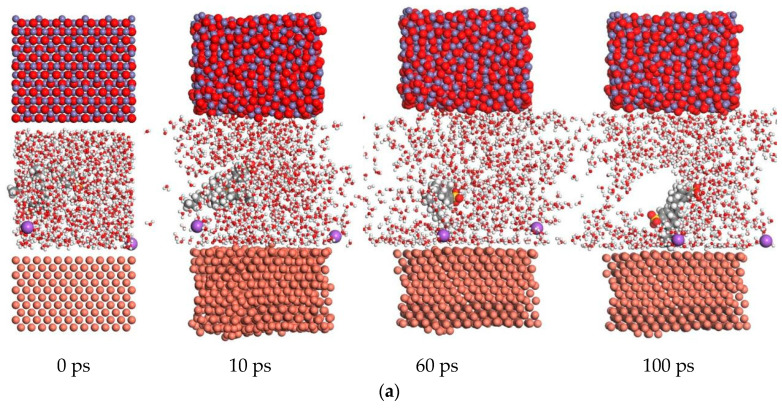

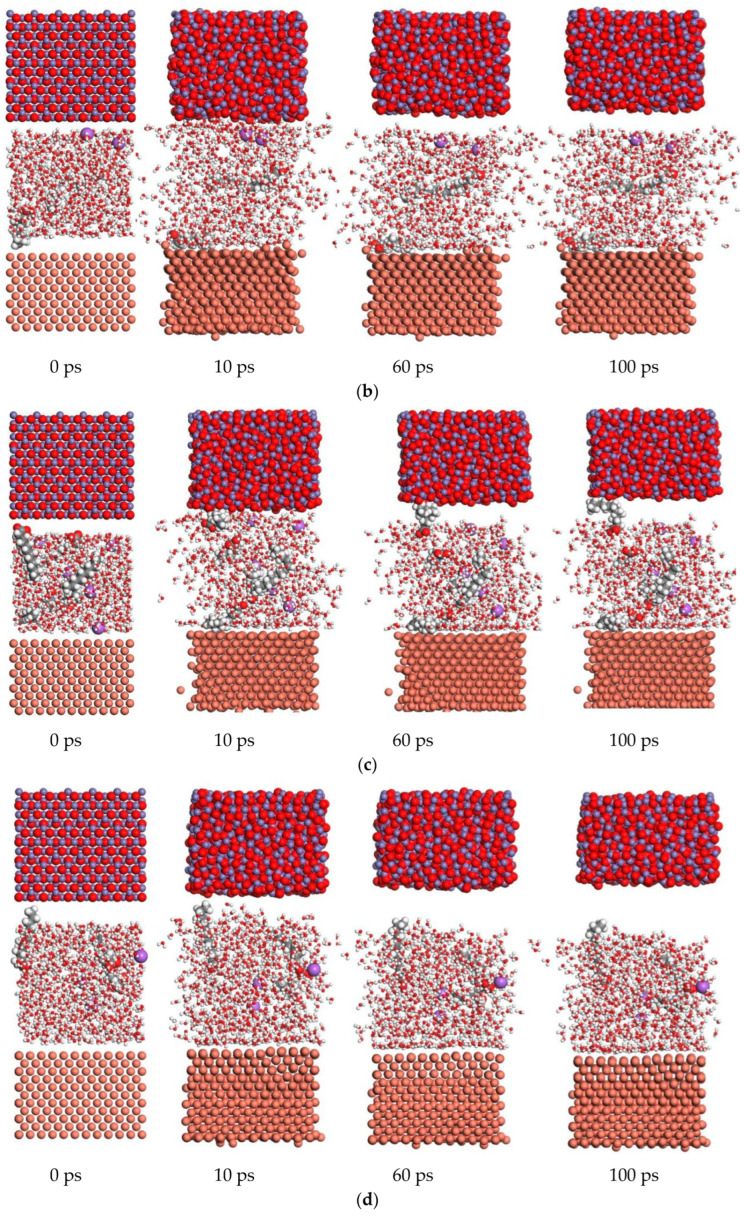
The dynamic molecule positions for different lubricants at different times. (**a**) The dynamic molecule positions of the SDBS nanolubricant. (**b**) The dynamic molecule positions of the sodium oleate nanolubricant. (**c**) The dynamic molecule positions of the mixed nanolubricant. (**d**) The dynamic molecule positions of the sodium laurate nanolubricant.

The dynamic paths of the molecules are shown in Figure 14 under high limiting temperature. The molecule position in the SDBS nanolubricant is shown in Figure 14a at different times. The absorption energy on the surface of the copper sample was mainly provided by water molecules at 498 K. The absorption power of the SDBS solution was lower than that of the other solution. The molecule position characteristic of the sodium oleate solution at different times is shown in Figure 14b. The long-chain molecules of the sodium oleate were found to have a significant effect on the absorption energy of the solution on the surface of Cu. The long-chain molecules were able to adsorb on the surface of the copper. The absorption power of the solution was more stable. However, the absorption power of the solution on the nanoparticles was insufficient. The water molecules were only dispersed on the surface of the Fe_3_O_4_ nanoparticles. The dynamic molecule positions for the solution containing sodium oleate and sodium laurate are shown in Figure 14c. The long-chain molecule always adsorbed on the surface of the copper sample. The absorption power was mainly provided by the long-chain molecule. At the same time, the long-chain molecule was adsorbed on the surface of the Fe_3_O_4_ nanoparticles. Nanoparticles could be stably dispersed on the surface of copper. The dynamic molecule positions of the sodium laurate are shown in Figure 14d at different times. The absorption energy on the surface of the copper sample was mainly provided by the water molecules. The absorption power remained constant. The surface of the nanoparticles did not adsorb the long-chain molecules. Nanoparticles are not easily dispersed on the surface of copper samples. In conclusion, when sodium oleate and sodium laurate were added to the water-based nanolubricant, a stable absorption performance by the lubricant was recorded. The lubricating properties of the nanolubricant can improve under different working temperatures.

**Figure 15:**
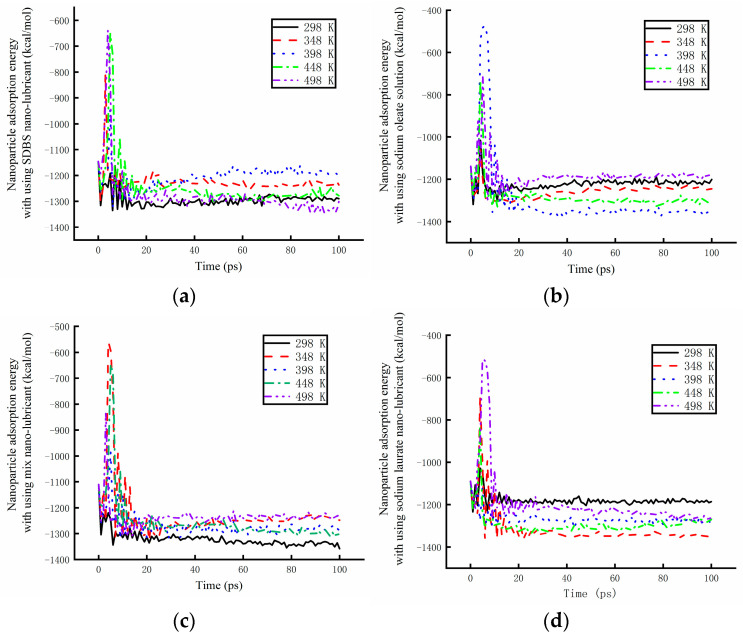
The absorption energy of nanoparticles in different lubricants under different working temperatures. (**a**) Nanoparticle absorption energy in the SDBS lubricant. (**b**) Nanoparticle absorption energy in the sodium oleate lubricant. (**c**) Nanoparticle absorption energy in the sodium oleate and sodium laurate lubricant. (**d**) Nanoparticle absorption energy in the sodium laurate lubricant.

The absorption energy of nanoparticles has a great effect on the performance of a lubricant. The absorption energy of Fe_3_O_4_ particles in different lubricants is shown in Figure 15. The absorption energy of particles in the SDBS lubricant is shown in Figure 15a. The absorption energy of particles reached its minimum at 298 K before 65 ps. The absorption power decreased at higher working temperatures. At 498 K, the best absorption power was obtained after 65 ps. The velocity of water molecules was incredibly high. The velocity of SDBS molecules increased. The SDBS molecules were easily captured on the surface of Fe_3_O_4_ nanoparticles after 65 ps. It was found that the absorption power of particles within the SDBS lubricant exhibits some differences under different working temperatures. The absorption energy of particles in the sodium oleate is shown in Figure 15b. The absorption energy was higher at 298 K, and the absorption power of particles was more unstable. With the increase in working temperature, the absorption energy significantly decreased, and a stable absorption power was obtained. However, at 498 K, the absorption energy reached its maximum, while the absorption power of particles reached its minimum. The performance of the absorption power became worse at different temperatures. The absorption energy of nanoparticles in the water-based lubricant containing sodium oleate and sodium laurate is shown in Figure 15c. At 298 K, the absorption energy reached its minimum, and the absorption power of the particles reached its maximum. The absorption power decreased with increasing working temperatures. However, the effect of temperature was lower than that found for other lubricating solutions, and the absorption power of the particles in the mixed lubricant became more stable. The absorption energy of nanoparticles in the sodium laurate lubricant is shown in Figure 15d. It can be seen that the absorption energy reached its maximum at 298 K, while the absorption power of the nanoparticles reached its minimum at the same temperature. When the working temperature was warm, the absorption energy decreased. The working temperatures have great effects on the absorption energy, and the absorption power of nanoparticles tend to become more unstable. In conclusion, the absorption power of nanoparticles in the mixed lubricant was found to be the most stable. The sodium oleate and sodium laurate were, therefore, added to the water-based nanolubricant, which was successively used for the processing of the copper sample in the four-high mill.

### 3.3. Rolling Test Results

The base solution was prepared for the processing of the copper sample, and the sodium oleate, sodium laurate, and deionized water were the main components of the base solution. The performance of the water-based nanolubricant was determined by conducting tribology tests. The lubricant containing 6 wt% Fe_3_O_4_ nanoparticles was found to deliver better tribological performances. The nanolubricant was also poured into the lubricating system of the four-high mill. At the same time, some copper samples were prepared for the rolling test, with a length of 90 mm and a thickness of 0.2 mm. 

**Figure 16:**
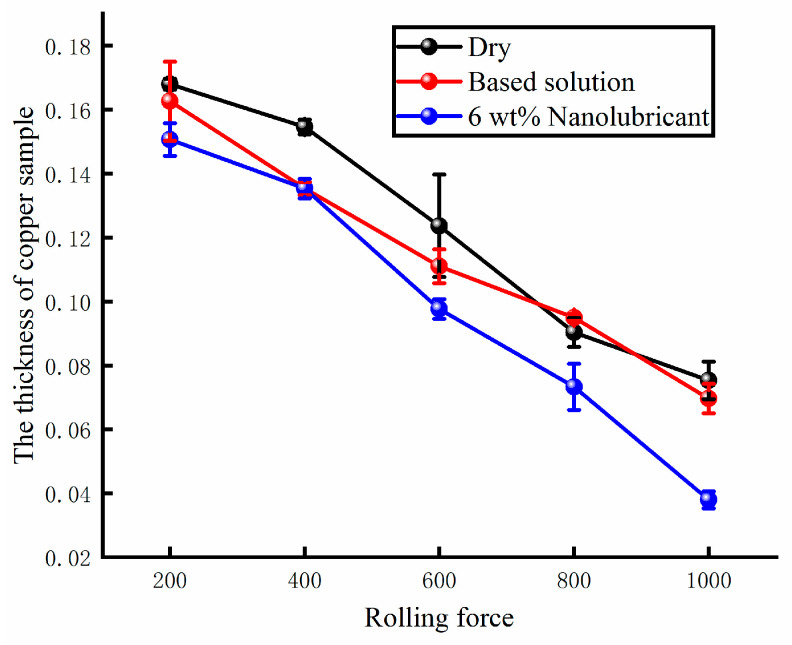
The thickness of the rolled Cu samples under different lubricating conditions.

The thickness of the samples is measured under different rolling forces. The thicknesses of the copper samples are shown in Figure 16 under different lubricating conditions. The thickness was found to decrease with increasing rolling force. The rate of thickness reduction was lower in the absence of lubricants. When the base solution was poured into the lubricating system of the four-high micro rolling mill, the rolling power improved under the lower rolling force. The effect of the base solution on the rolling thickness decreased under the higher rolling force. The 6 wt% nanolubricant was used for the rolling tests. The rolling thickness became thinner under the same rolling force. Under different rolling forces, the rolling thickness of the sample experienced an improvement as a result of the nanolubricant. The thinnest samples were obtained by using the nanolubricant. Meanwhile, the required rolling energy decreased when using the 6 wt% water-based nanolubricant.

**Figure 17:**
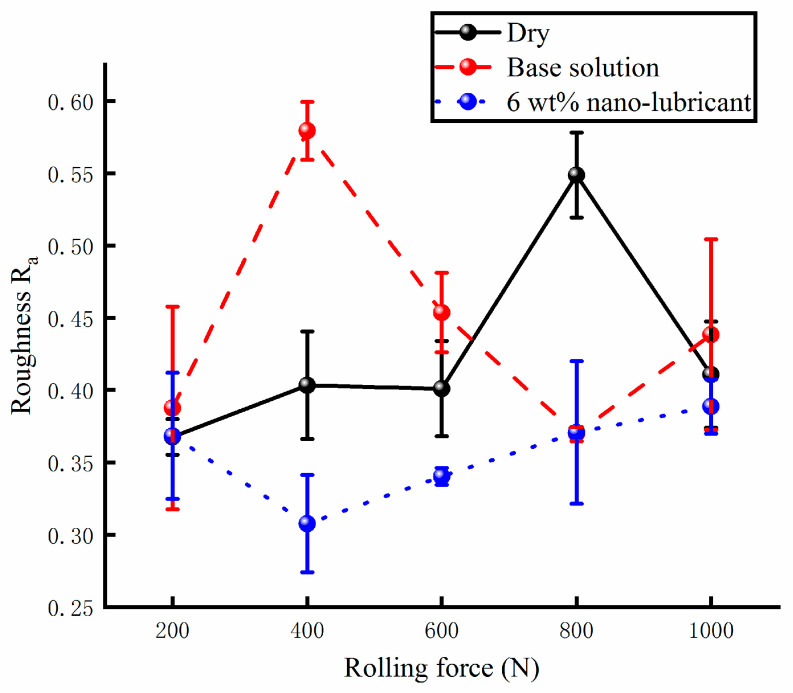
The surface roughness R_a_ of rolled strips under different lubricating conditions.

The processing quality of the rolled Cu strips were measured using a surface roughness meter. The surface roughness of the samples is shown in Figure 17. It can be seen that the change in surface roughness was unstable when measured in the absence of lubricants. The maximum value of surface roughness reached was about 0.55. The effect of the base solution was also tested. The change in surface roughness was unstable. The maximum value of R_a_ reached was 0.6, while the minimum value of R_a_ reached was 0.32. The processing quality of the samples was not found to improve following usage of the base solution. However, when the 6 wt% nanolubricant was poured into the lubricating system of the micro rolling mill, the change in surface roughness became stable. The value of R_a_ was found to be lower than that of documented under the other lubricating conditions. The processing quality of the rolled Cu samples was found to improve when the water-based nanolubricant was used.

**Figure 18:**
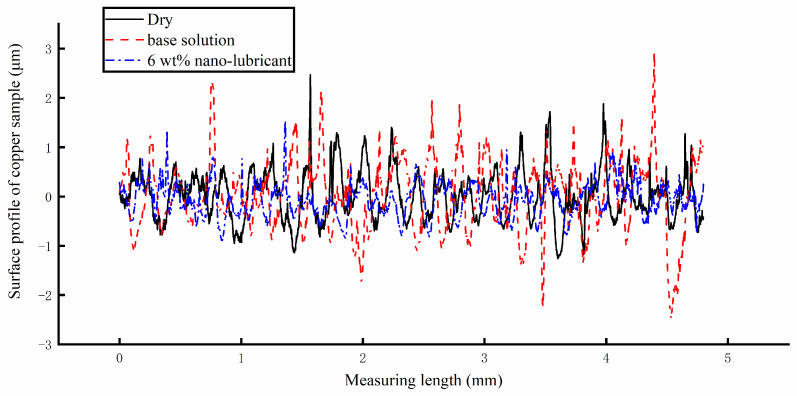
The surface profile of the rolled Cu strips under different lubricating conditions.

The surface profiles of the Cu strips were measured using a surface roughness meter under a 400 N rolling force, with the results shown in Figure 18 under different lubricating conditions. It can be seen that the direction of the tested distance is perpendicular to the rolling direction. The tested distance was set to 5 mm. The wave range of the surface profile when using the water-based nanolubricant was lower than that recorded under the other lubricating conditions. The maximum surface profile was about 1 μm, while the minimum was about −1 μm. Without using any lubricants, the maximum surface profile was found to be over 2 μm. When the base solution was poured into the lubricating system of the micro rolling mill, the maximum and minimum values with respect to the surface profile were found to be both over 2 μm. Therefore, the water-based nanolubricant can improve the surface quality of the rolled sample.

**Figure 19:**
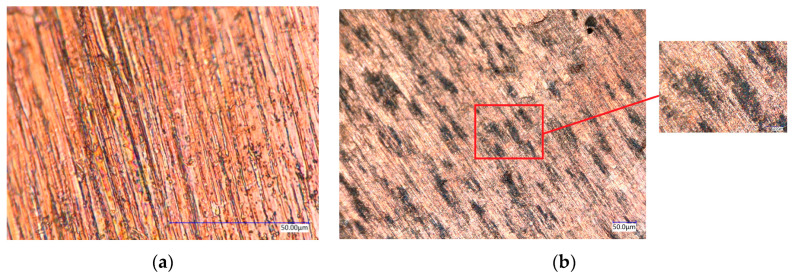
The surface morphology of the rolled samples under different lubricating conditions. (**a**) Surface morphology of Cu samples in the absence of lubricants. (**b**) Surface morphology of Cu samples using the 6 wt% water-based lubricant.

The surface morphology was filmed using a microscope, as shown in Figure 19. It can be seen that the surface morphology of the rolled sample obtained in the absence of lubricants was very coarse (Figure 19a). When the water-based nanolubricant was used, the surface quality improved (Figure 19b), and the bumps and pits found on the surface significantly decreased. In this case, the nanoparticles were able to fill in some pits. In the rolling tests, the nanoparticles essentially acted as grinding tools, and some bumps found on the copper surface were worn down by the particles; the surface of the roll was protected by the nanoparticles.

## 4. Conclusions

A new method for the preparation of water-based nanolubricants was investigated as part of this study. The nanoparticles of Fe_3_O_4_ were added to the base solutions to improve the friction performance of the lubricants. The surfactants of particles have significant effects on the anti-friction performance of lubricants. The results show that the water-based nanolubricant is a safe and reliable lubricant, and the proposed method is simple and associated with production costs that are lower than those typical of other processing methods. The water-based nanolubricant can be widely used for the rolling of stripes in the future. The oil-based processing lubricant will be replaced. The process of rolling strips will become more environmentally friendly. However, the lubrication mechanism of water-based Fe_3_O_4_ nanolubricant is very complicated. We are confident that the problem could be solved with future research.

When 20 nm particles were mixed into the base solution, the film strength of the lubricant significantly improved. In the long-time test, the lubricants mixed with 6 wt% and 8 wt% of Fe_3_O_4_ particles exhibited better anti-friction performance, and the 6 wt% water-based nanolubricant delivered the best tribological performance.

The surfactant of particles is an important element in the preparation of nanolubricants, as the surfactants have effects on the absorption energy of the lubricant. The absorption power is also an important parameter. When sodium oleate and sodium laurate were both added to the solution, the absorption energy was found to become more stable. Therefore, sodium oleate and sodium laurate were both selected as the best lubricating additives for the preparation of the water-based nanolubricant.

The rolling test showed that, under the same rolling force, the thickness of Cu samples was thinnest when using the 6 wt% water-based nanolubricant. The surface quality also improved when using the 6 wt% water-based nanolubricant. 

## Figures and Tables

**Table 1:** The atom labeling in simulation.

Atomic Type	Atomic Color in the Numerical Simulation
Carbon atom	Gray
Hydrogen atom	White
Oxygen atom	Red
Sulfur atom	Yellow
Iron atom	Purple
Copper atom	Orange

## Data Availability

The data that support the findings of this study are available on request.
